# Obituary

**Published:** 1910-02-12

**Authors:** 


### Obituary.

We have to announce the death of Mr. Samuel Evans, of Harwich and Dovercourt, at the age of sixty-three. Mr. Evans studied at University College, and took his diploma in 1870. He had retired from practice at the time of his death.

The death is announced of Deputy-Surgeon-General Robert Rouse, I.M.S., at the age of seventy-seven. He had retired from the Service and had been attached to the Frontier Force in India; on his return to England he lived at Reigate.

The death, at Karonga, Nyasaland, British East Africa, is reported of Mr. Arthur Frost Forster, Medical Officer to H.M. Government. Mr. Forster, who had been a student at St. Bartholomew's, took his diploma in 1904, and was a member of the British Medical Association.

